# Supplementary figures and images for: Capture and visualization of live Mycobacterium tuberculosis bacilli from tuberculosis patient bioaerosols
Source: PLoS Pathog. 2021 Feb 1;17(2):e1009262. doi: 10.1371/journal.ppat.1009262 (PMC7877778; doi:10.1371/journal.ppat.1009262)

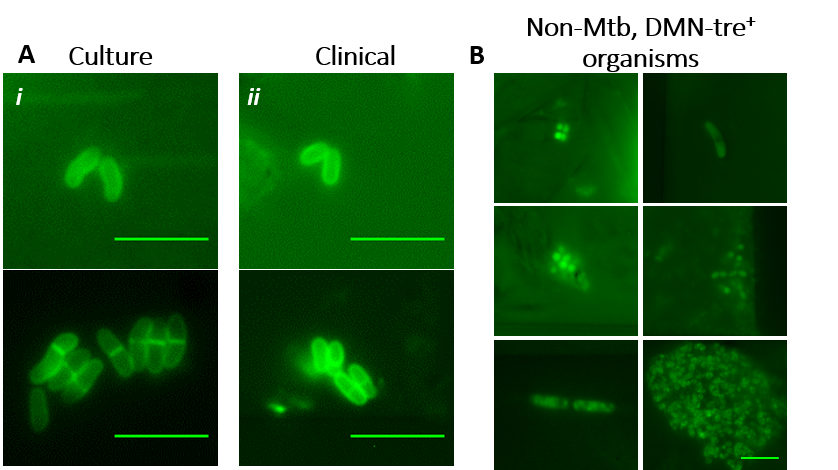

Supplement: S1 Fig — (A) Representative images of (i) C. striatum cultured in LB broth during log-phase and stained with 100 uM DMN-trehalose for 5 min, and (ii) DMN-tre+ organisms detected within a bioaerosol sample. (B) A panel of non-Mtb, DMN-tre+ organisms, as determined by our inclusion criteria, identified in various bioaerosol samples. Scale bar, 5 μm. (TIF) [file ppat.1009262.s001.tif]

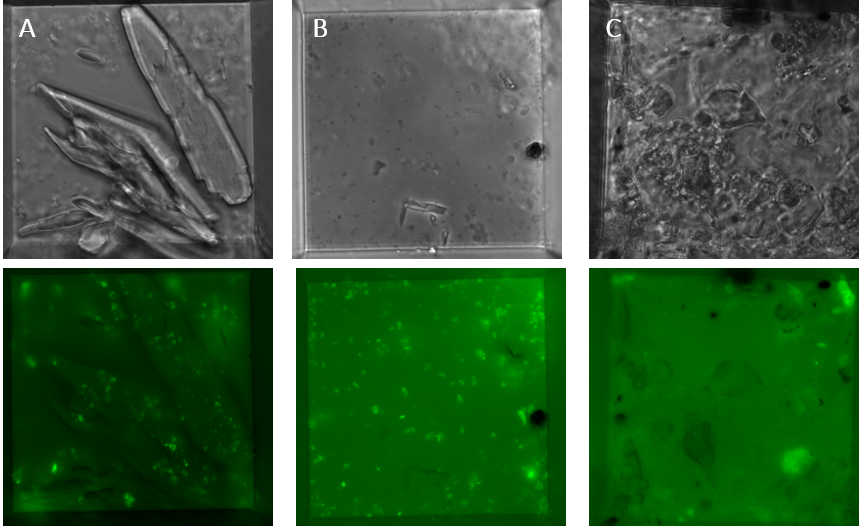

Supplement: S2 Fig — Representative images of the three major categories of debris found within bioaerosol samples after overnight staining with 100 μM DMN-trehalose and visualization within a 50 x 50 μm nanowell. (A) Large, crystalline debris, (B) small fluorescent debris, and (C) granular debris. (TIF) [file ppat.1009262.s002.tif]
