# Supplementary material for: Capture and visualization of live Mycobacterium tuberculosis bacilli from tuberculosis patient bioaerosols
Source: PLoS Pathog. 2021 Feb 1;17(2):e1009262. doi: 10.1371/journal.ppat.1009262 (PMC7877778; doi:10.1371/journal.ppat.1009262)
Supplement: Attachment — Submitted filename: DINKELE GESSNER et al_RESPONSE TO REVIEWERS.docx [file ppat.1009262.s004.docx]

**RESPONSE TO REVIEWERS: PPATHOGENS-D-20-01425**

**Part I – Summary**

***Reviewer #1***:

This paper presents very novel and potentially exciting methods and data, but unfortunately it has many weaknesses that the authors do not acknowledge. I strongly recommend the inclusion of a 'limitations' section of paragraph towards the end.

*⮚ Response*: We thank the reviewer for these encouraging comments and accept that the limitations were inadequately acknowledged in the original submission. To address this valid criticism, a paragraph detailing the limitations of this work has been included in the revised manuscript (Lines 316-44).

My major concern is the lack of any culture data on the patient’s samples, in spite of the title suggesting that there is capture of 'live *M. tuberculosis*.' Ideally it would have seemed reasonable to compare the bioaerosol collected to that collected by an Andersen cascade impactor, as used in other studies. But at minimum the collection from the Coriolis sampler could have been cultured. Why was this not done?

*⮚ Response*: The detection of DMN-trehalose fluorescence-positive organisms supports the claimed “live capture”. Nevertheless, the reviewer’s criticism is valid and reflects concerns expressed elsewhere by this and the other reviewers relating to specificity. The central challenge informing our handling of bioaerosol samples is that the extreme paucibacillary nature of the samples (our median counts are in the range 10^0^-10^1^ organisms) means that splitting samples is impossible owing to the risks of effectively diluting numbers even further, while sequential analyses in bulk (such as the microbiological culture proposed here) demand the ability to recover bacilli arrayed on the nanowell devices (technically enormously challenging in the absence of microfluidic capabilities) for inoculation into a culture tube (MGIT, *etc*.). Moreover, culture of organisms eliminates the benefit inherent in the arrayed device, namely that bulk culture in the absence of compartmentalization risks outgrowth of contaminating organisms that will obscure the *Mtb* signal. Finally, even when accomplished, “successful” culture of *Mtb* cannot be directly linked to the “putative *Mtb*” bacilli identified microscopically; that is, any inference is unavoidably indirect. For these reasons, we continue to prioritize the culture-free identification of *Mtb* from aerosol samples.

As acknowledged in the new “limitations” paragraph (Lines 316-44, see comment above), although we have not presented orthogonal evidence confirming the identity of the “putative *Mtb*” identified microscopically, we respectfully contend that the presumptive evidence is strong:

(i) The analyses presented here are limited to bioaerosol samples that were obtained from GeneXpert-positive TB patients immediately after diagnosis and before treatment initiation.
(ii) Previous results from our research group have demonstrated the isolation in the RASC platform of PCR-confirmed *Mtb* colony forming units in bioaerosol samples (DOI:10.1371/journal.pone.0146658; DOI:10.12688/gatesopenres.12758.2).
(iii) In ongoing work (these data are neither included nor referred to in the revised manuscript and are mentioned here solely because they are relevant to the reviewers’ comments), two sequential bioaerosol samples collected from the same patient are required to undergo analysis via DMN-trehalose probing and either Auramine O fluorescence microscopy or RD9 PCR detection. These analyses, applied to separate subsets of a single GeneXpert-positive patient cohort, have confirmed 100% positivity correlation (albeit that the absolute counts differ). A summary of these preliminary results is provided in the table below:

(iv) In work initiated recently (these data are neither included nor referred to in the revised version because the study is in its infancy), we have started to investigate the utility of bioaerosol sampling to monitor the response to TB treatment. To date, we have enrolled a small cohort of 9 diagnosed TB patients: 8 sputum GeneXpert positive and 1 sputum GeneXpert-negative. Prior to initiation of therapy, putative *Mtb* organisms were identified in all 9 patients with a median count of 24 (IQR 18-37). To determine the treatment effect, 6 patients were retested at two weeks and 2 months post standard four-drug combination therapy. Bacillary counts decreased significantly at two weeks (p=0.04) to a median value of 15 (IQR 12.5-16) and at 2 months (p=0.004) to a median value of 6.5 (IQR 4.2-8.5). Again, these data might be considered circumstantial, however we have mentioned them in this rebuttal as we are encouraged by our ability to detect this decline in “putative *Mtb*” numbers as a function of treatment duration.

The use of DMN trehalose has only been done on sputum samples and not previously on aerosols, which may contain bacilli that are more stressed and may not have the same responses as those in sputum.

*⮚ Response*: This is a valid point. We agree that the morphological and other characteristics of sputum *Mtb* might differ from aerosol *Mtb* and that this could impact DMN-trehalose incorporation profiles. In fact, this possibility provided partial motivation for our switch to cytological profiling-based approaches to characterize bacilli isolated from different clinical samples. In the new “limitations” paragraph, we acknowledge that the criteria we used to classify DMN-tre^+^ organisms as “putative *Mtb*” are potentially restrictive, especially given that prevailing assumptions about the size and shape of clinical *Mtb* isolates are heavily influenced by the commonly applied staining methods (incidentally, we note that the “accepted” staining methods are almost never supported by confirmatory molecular or microbiological data). We are also cognizant of the bias which we all hold and is based on our knowledge of *Mtb* morphology from growth *in vitro* in defined culture, in some cases in intracellular infection models or under applied stress conditions. There is a strong likelihood that we are failing to detect *Mtb* which do not conform to these criteria and, moreover, our use of fluorescence positivity necessarily excludes organisms which might be transiently inactive or quiescent. As noted in the revised text, ongoing and future work is exploring the incorporation of automated image detection software to facilitate machine-driven detection of “interesting” structures following capture of microscopy images for all particulate matter (organic and inorganic) arrayed on the nanowell slides. This development is proposed to address a further limitation of our approach, namely that our method relies on detecting then imaging cells (including microscope focusing) based on DMN-trehalose fluorescence. The noise in some samples means finding an optimal focus can be a challenge, which may artifactually increase or decrease the width (and to some degree the length) of the bacilli we measure. Finally, we have observed that aerosol samples are generally “cleaner” than the corresponding sputum samples from the same patient; therefore, it is quite possible that the limitations inherent in DMN-trehalose use are greater for sputum than bioaerosol.

In a similar vein, even though the RASC helps reduce mold contamination from the outside environment, cough aerosols have contained other organisms.

*⮚ Response*: Another valid point. However, we respectfully submit that the argument outlined in the comment above addresses this concern. Moreover, as we have consistently maintained in developing the RASC technology, we have not focused on cough aerosols, and instead collect bioaerosol produced within an hour which may contain cough-derived aerosols, too, but this often unlikely to constitute the as major constituent.

In the original cough aerosol paper (Fennelly et al, 2004), nontuberculous mycobacteria, diptheroids, Cladosporium and other Gram-positive and Gram-negative pathogens were isolated from the patients. How can you confirm that the DMN trehalose stain is so specific that it is not staining some of the patients' other respiratory flora?

*⮚ Response*: This is a fair comment; in fact, the pioneering work of Fennelly and colleagues has strongly influenced our own studies. Since this criticism reiterates the reviewer’s earlier concerns about specificity, we hope that the responses offered above have addressed this comment adequately.

The study population is poorly characterized. Patients who are Xpert-positive are likely, but not always culture positive. Given the traditional use of AFB smears and cultures, these simple tests would have helped characterize the population better for readers. It is also usually customary to include some description of imaging, at least whether or not there was cavitary disease.

*⮚ Response*: As acknowledged in the “limitations” paragraph (Lines 316-44), it is true that the bacillary counts and morphological phenotypes presented here are not augmented with clinical metadata (chest radiography scores, HIV status, *etc*.). Our intention in this study was to establish the technological platform for bioaerosol sample capture and analysis. We recently published a protocol (DOI:10.1186/s12879-020-05278-y) describing the planned application of the RASC platform to carefully designed clinical cohorts. The reviewer nevertheless makes an important point about the common discordance between GeneXpert-positivity and culture-positivity. In fact, this issue – which is the subject of intense research – highlights a central challenge in the TB field: that our view of the disease is framed within (even constrained by) the available tools. In developing the RASC, we have prioritized culture-independent detection of metabolically active (not necessarily replicatively active, nor “culturable”) bacilli, precisely to enable greater insight into the number and nature of potentially infectious *Mtb* organisms that might drive TB prevalence. In our research group, we are moving steadily towards our longer-term goal which is to link the phenotypes (and genotypes) of the organisms we detect to successful transmission events; this manuscript represents another critical step in demonstrating the capacity of the technology to enable live cell capture and non-invasive detection and characterization of aerosol *Mtb* in single-cell format.

In line 202, reference 17 is not the best source for the statement, as that paper does not include anything about infectiousness or transmission. That statement can also be challenged, as one study found that cough aerosol cultures of Mtb were the best predictors of infectiousness and transmission of new infections to household contacts (Jones-Lopez et al, 2013). In addition, ref #17 is not the best choice in line 103, as the method used was originally published by Honeybone in the J Clin Microbiol in 2011.

*⮚ Response*: The reviewer’s point is well made. However, the section on profiling of *Mtb* bacilli in sputum has been removed from the revised manuscript, obviating the need to replace the inappropriate reference.

***Reviewer #2:***

The paper by Dinkele and colleagues details new methods for the detection of viable Mycobacterium tuberculosis in bioaerosols. The controlled nature of the air sampling procedure, coupled with a novel use of a fluorogenic substrate as a vital stain, makes this study extremely valuable to the field. The major finding, that the release of viable organisms, is significantly greater than were estimated by previous sampling methods, is important to the field. Moreover, this protocol has several potential applications linked to therapeutic interventions as a much more informative and sensitive “time to sputum negativity” type assay.

*⮚ Response*: We are grateful to the reviewer for the generous endorsement of our approach. As the reviewer notes, the potential applications are broad and significant – our task is to develop a reproducible and reliable technology that can be applied as envisaged.

***Reviewer #3:***

Dinkele, Gessler et al. report the development of a novel device (a nanowell arrayed microscope slide) which, when combined with staining by a solvatochromic dye (DMN-trehalose) allows quantitative visualization of exhaled Mtb from patient bioaerosols. The respiratory aerosol sampling chamber (RASC) has been previously described (PLoS One 2016) and the sensitive capture of Mtb (as identified by culture and/or PCR) from patient bioaerosols was reported in 2018 (Gates Open Research). The current publication introduces the nanowell capture device paired with the application of the DMN-trehalose dye and shares the morphologic and staining characteristics of the captured organisms.

While the reviewer is in principle strongly supportive of aerobiology research which s(he) views as pioneering work on the frontiers of modern microbiology, the data included in the manuscript are descriptive, without definitive link to mechanism or novel insight into pathogenesis. Inclusion of downstream analysis (whole genome sequencing, proteomics, metabolomics, other), from even only a few patient aerosol samples would suffice to increase interest in publication to inform and inspire the broader microbiology community.

*⮚ Response*: The reviewer makes an excellent point and, in response to this comment, we pursued the experiments which are described in the new section included under Results (L214-37, new Fig. 5) in the revised manuscript. This work describes the serial imaging over two weeks post identification of single “putative *Mtb*” bacilli detected in the nanowell device. Although we have not yet detected gross morphological changes, let alone replication, we respectfully submit that this represents a significant technical advance in enabling the kind of analyses which the reviewer suggests (and in which we are equally invested).

**Part II – Major Issues: Key Experiments Required for Acceptance**

***Reviewer #1:***

My comments above might be interpreted to say that culture from the Coriolis should absolutely be done prior to acceptance. I suspect that may be impossible at this point. There is enough exciting potential in these methods that i would hate to see this not published at all. But the authors clearly need to describe the limitations in the paper and to explain some of the reasons for not doing some of what I suggested.

*⮚ Response*: As indicated above, we are very grateful to the reviewer for these positive comments and apologize that the limitations were inadequately acknowledged in the original submission. To address this criticism, we included a new “limitations” paragraph (lines 316-44) which has been referred to throughout this rebuttal. In addition, we hope that we have provided acceptable explanations for and/or the rationale informing our perceived failure to undertake some of the experiments suggested.

The sentence from line 105 to 108 is very confusing and needs re-writing for clarity. Although cough obviously assumes symptomatic spread, cough has been shown to be associated with 'natural transmission' by both R. Loudon and R. Turner.

*⮚ Response*: DONE. The sentence has been re-drafted as suggested.

In addition to addressing the limitations mentioned above, in your concluding paragraph of the discussion, it would be helpful to address how you plan to link these findings to transmission, e.g. in a household contact study?

*⮚ Response*: DONE. A new concluding paragraph (L348-62) offers some ideas for future applications of this technology.

***Reviewer #2:***

The paper is written in a clear and well-balanced manner, the data are described with care and rigor, and the short-comings and caveats with respect to specificity and heterogeneity are discussed fully. One could ask for more data to be included but I feel this would not alter the basic findings or the immediate utility of this method. I believe it more important that these findings should be published quickly.

*⮚ Response*: Again, we thank the reviewer for these very encouraging remarks and hope that, while no formal request was made for specific revisions, the modified version submitted here has addressed some of the concerns alluded to in this comment.

***Reviewer #3:***

Lines 34-35, although it is correct that ‘the contribution of asymptomatic transmitters to the TB pandemic is overlooked’ this is not addressed by the data presented in the manuscript and should be removed from the abstract. It however represents a valid and important area of future research that is appropriately included in the discussion.

*⮚ Response*: DONE. We have revised the Abstract accordingly.

Lines 43-44 ‘variations in DMN-trehalose incorporation suggested metabolic heterogeneity in aerosolized Mtb’ the heterogeneity depicted in Figure 4 is difficult for the reviewer to perceive.

*⮚ Response*: The reviewer’s comment is well made. Figure 4 has been revised in order to improve the clarity of the images and associated analyses.

Can more distinct images be included? Can the heterogeneity be quantified via imaging software and statistics applied to confirm the presence of three distinct phenotypes? In addition, authors attribute heterogeneity in DMN-trehalose staining to metabolic state; inclusion of DCTB staining or staining of organisms undergoing in vitro antimycobacterial drug exposure for direct comparison would more strongly support this claim (Kamarizza Sci Trans Med 2018, Figure 7)

*⮚ Response*: DONE. We thank the reviewer for this suggestion, which motivated the inclusion of new Figures 2 and S1, and revised Figure 4. In addition, current and planned work will include the use of antibiotic exposures *in vitro*, and a panel of different fluorescent probes (*e.g*., nucleic acid dyes such as DAPI and the SYTO series, and cell envelope probes including the fluorescent D-amino acids) to develop a more complete database for cytological comparisons.

Lines 44-46 ‘intrapatient comparisons indicated that Mtb bioaerosols were probably derived from a compartment other than sputum’. This is conjecture and not well-supported by the data which show only a minor length difference between bioaerosol-derived and sputum-derived organisms. This difference could be due to sampling method as Coriolis subjects bioaerosols to manipulation that differs from standard sputum sampling. This claim requires additional data to support (whole genome sequencing, metabolomics, proteomics) or, further clarification of the basis upon which the claim is made.

*⮚ Response*: The reviewer makes an excellent point. In the absence of sufficiently large sample sizes to support the original inference, and given the unavoidable reliance on centrifugal concentration for capture of aerosol organisms via the liquid cyclone, we determined the most prudent approach was to remove all data and text pertaining to sputum versus aerosol *Mtb* in revising the manuscript.

Line 164, the major finding of the manuscript is that staining with DMN-Tre (89% positive) closely approximates the previously published culture/PCR positivity rates in exhaled bioaerosols from TB patients (93%, Gates Open Research 2018) while still enabling down-stream analysis. Some evidence of that downstream analysis (whole genome sequencing, metabolomics, proteomics or other) should be included in the manuscript as the reported findings otherwise represent an important but incremental advance beyond the prior publications describing the RASC (Plos One 2016) and the capture of viable Mtb from patient bioaerosols (Gates Open Research 2018) which in and of itself is not of broad interest to the wider microbiology community.

*⮚ Response*: As noted above, this is a valid point, and prompted us to undertake the serial imaging experiments described in the new Results section (L214-37). We acknowledge that imaging alone does not satisfy the request for an ‘omic analysis of the captured organisms, however we trust that the reviewer will agree that the capacity for extended incubation and analysis constitutes the type of advance that will be of greater general interest, in particular in demonstrating our proximity now to having the technological platform in place that can accommodate the investigations proposed.

**Part III – Minor Issues: Editorial and Data Presentation Modifications**

*Reviewer #1*:

Line 67, the ref #1 should be placed at the end of that sentence.

*⮚ Response*: DONE.

Line 108: there is no period at the end of the sentence.

*⮚ Response*: DONE.

Line 134: 'bespoke' is a rather obscure word. Why not say something more direct, e.g. 'custom'?

*⮚ Response*: DONE. We have replaced “bespoke” with “custom-designed”

Reviewer #2: (No Response)

Reviewer #3:

Might alter the title to “Capture and visualization of live Mycobacterium tuberculosis bacilli from patient bioaerosols”

*⮚ Response*: DONE. The title has been changed accordingly, with the qualifier “tuberculosis patient bioaerosols” to indicate that the results presented here are restricted (for now) to GeneXpert-positive TB diagnoses.

Lines 51-53 ‘ongoing transmission…is the primary driver of incident disease’. Important to provide citation here as this may well be true in high transmission settings in Sub-Saharan Africa but is controversial in other geographies, for example China, where some models support reactivation as the driver of the epidemic.

*⮚ Response*: This point is well made. We agree with this distinction; however, in re-writing the “Author Summary” we have eliminated this sentence.

Lines 53-57 include citation here as well. this paragraph reads very similarly to Andrews ERJ 2019 which shares co-authors with this manuscript. This may well be due to the nature of introductions about TB transmission but authors should take care not to repeat themselves too closely.

*⮚ Response*: We thank the reviewer for pointing this out, and apologize for the overlap. The paragraph has been almost entirely rewritten to reduce the extent of similarity with the cited work (L54-68).

Line 65 interventions “to better understand and ultimately interrupt Mtb transmission”?

*⮚ Response*: We thank the reviewer for this suggestion. The final sentence of the Author Summary has been modified to include this phrase.

Line 146 please address the differences in width in the discussion section (what is the likely source and significance of this, if any)?

*Response*: In preparing the revised manuscript, we felt that our sample numbers were too low to make claims about morphological differences between *Mtb* bacilli obtained from different sources (*in vitro* versus *in vivo*, inter- and intra-patients variability, *etc*.). Therefore, to minimize the risk of poorly substantiated claims about characteristic “features”, this element of the results and discussion was removed. Nevertheless, the new “limitations” paragraph (L319-47) does partially address this point in highlighting the challenges faced in microscopic analyses of aerosol samples.

Figure 2C has both columns i and ii as well as two rows; the columns are labelled but the rows are not; how does the top row complement or differ from the bottom row?

*⮚ Response*: We apologize for the confusion. The modified version of this figure now appears as Figure S1 in the revised manuscript.

Figure 2D is not described in the text; which non-Mtb, DMN-tre+ organisms are in which row and column? This should be clarified in the legend.

*⮚ Response*: We apologize for the confusion. The modified version of this figure now appears as Figure S1 in the revised manuscript.

Line 162 please confirm that the 28 patients reported here are not a subset of the 35 patients previously presented in the 2018 Gates Open Research publication.

*⮚ Response*: Confirmed. The data contained in this manuscript derive from a separate patient cohort that has not been described previously.

Figure 3 image ii (patient) makes more sense first followed by image i (RASC).

*⮚ Response*: We thank the reviewer for this suggestion, Figure 3 has been revised accordingly.

Are there any differences in any parameter based on strain lineage?

*⮚ Response*: This is an interesting question. We do not have enough data currently to answer this definitively.

Table 1, what is meant by NA in the table? Please add a footnote clarifying this.

*⮚ Response*: Table 1 has been revised to exclude all patient data.

HIV+/HIV- seem not to differ in Mtb counts; this is unexpected (Kwan and Ernst Clin Micro Rev 2011) and should be addressed in the discussion

*⮚ Response*: This data presented here are not powered to address questions around specific patient characteristics such as gender, HIV status, smoking status, etc. Therefore, to eliminate the potential for erroneous conclusions based on an inadequate sample size, Table 1 has been revised to exclude all patient data.

Lines 168-173 as noted above (abstract) the three patterns are indistinguishable to the reviewer. Please provide higher resolution images and quantitative analysis; also please include an image of PBS-starved organisms for direct comparisons (currently the reader has to ‘take your word for it’).

*⮚ Response*: DONE. Please see the revised Figure 4.

Line 175 please include more detail in the discussion about the potential significance of patient-derived organisms being shorter than organisms in log phase growth**.**

*⮚ Response*: As noted above, in preparing the revised manuscript, we felt that our sample numbers were too low to make claims about morphological differences between *Mtb* bacilli obtained from different sources (in vitro versus in vivo, inter- and intra-patients variability, etc.). Therefore, to minimize the risk of poorly substantiated claims about characteristic “features”, this element of the results and discussion was removed. Ongoing work aims to increase significantly the total number of *Mtb* bacilli collected and analysed.

Lines 179-182 please include data demonstrating non-Mtb DMN-Tre+ organisms in a supplemental figure

*⮚ Response*: DONE. Please see Figure S1.

Line 186 because no physical or biochemical analysis of particulate matter was performed, Figure 5 should be relegated to a supplemental figure and the text shortened accordingly; if, in contrast such analysis exists it could be included in the main text (identification of host-associated molecules, mucus or metabolites for example)

*⮚ Response*: DONE. The original Figure 5 has been moved to Figure S2.

Lines 210-215 why are only 3 of the 4 patients shown? Very difficult to observe polar staining differences and therefore would benefit from inclusion of a summary figure as is included for length. Also the potential mechanisms underlying differences in growth/polar staining between cultured organisms and patient bioaerosol should be more clearly elucidated in the discussion.

*⮚ Response*: We apologize for the confusion arising from our including multiple images from the same sample. Nevertheless, given the decision (noted above) to remove all images and all text relating to the sputum versus bioaerosol comparison, this issue has been eliminated.

Line 231 results section suggests that some of the particles arise from Tyvek suits; because analysis of non-microbial constituents is not reported it is not possible to determine whether these particles arose from patient breath or other non-respiratory compartment that is of little relevance to TB pathogenesis

*⮚ Response*: We agree, and have removed this speculation in preparing the revised manuscript.

Lines 240-246 do not make sense to the reviewer. How can length be directly linked to cavity surface as the source of the organism? This conclusion seems to require substantially more evidence than is included in the manuscript.

*⮚ Response*: This point is well made. As noted above, we have removed images and all text relating to the sputum versus bioaerosol comparison.

Lines 293-296 “closing the loop” is unclear. Could bioaerosols be used to infect guinea pigs as a measure of infectiousness? Reference could be made to aerobiology studies from the 1890s (comprehensively reviewed by Peter Donald in an NIH webinar <http://www.stoptb.org/assets/documents/news/TCRB%20Presents.pdf> and manuscript published in IJTLD 2018). The last sentence could reference multiomic approaches to be applied to viable organisms isolated on a single cell basis directly from patient aerosols (proteomics, metabolomics, whole genome sequencing) which represent the frontier of modern microbiology.

*⮚ Response*: DONE. We thank the reviewer for this suggestion which has informed the text of the new concluding paragraph (L348-62)

References

Line 369, too many spaces between commas

Line 419, immunopathology in ?

Line 380, Articles?

*⮚ Response*: DONE. We apologize for these errors and thank the reviewer for alerting us to them.

Figure 3, vii the include/exclude distinction in the figure is uninterpretable, please include clearer examples and clarify rationale in the text (if software based, how was the software trained?)

*⮚ Response*: DONE. All classifications are visual using the criteria described in the text. Examples are included in new Figure S1.

Figure 4, what proportion of patients exhaled clumps? Was there a correlation with any clinical parameter? What proportion of total organism burden per patient with clumps was exhaled in clumps versus single organisms? What is the likely source and significance of this? What are the implications for measurement of drug response? Figure 4d what is the significance of cultured organisms being longer than aerosol? Why do you think this is the case?

*⮚ Response*: Data indicating the (in)frequency of clumps have been included (L205-7), and the potential significance of clumped bacilli is briefly explored in the Discussion (L249-51). We agree this is a potentially important observation; however, we respectfully contend that further work is required before we can speculate comfortably about the potential source and significance of this result.
